# Analyzing Volatile Compounds of Young and Mature *Docynia delavayi* Fruit by HS-SPME-GC-MS and rOAV

**DOI:** 10.3390/foods12010059

**Published:** 2022-12-22

**Authors:** Yun Wang, Yuheng He, Yun Liu, Dawei Wang

**Affiliations:** 1 Key Laboratory of Forest Resources Conservation and Utilization in the Southwest Mountains of China Ministry of Education, Southwest Forestry University, Kunming 650224, China; 2 Forest Resources Exploitation and Utilization Engineering Research Center for Grand Health of Yunnan Provincial Universities, Kunming 650224, China

**Keywords:** *Docynia delavayi*, volatile compounds, fragrance properties, HS-SPME-GC-MS, rOAV

## Abstract

This study focused on the examination of the volatile compounds and fragrance properties of the young and mature fruit of *Docynia delavayi*. Headspace solid-phase microextraction combined with gas chromatography–mass spectrometry (HS-SPME-GC-MS) was applied for identifying 42 volatile compounds, with young and mature fruit containing 36 and 42 compounds, respectively. Heat map cluster analysis, principal component analysis (PCA), orthogonal partial least squares discriminant analysis (OPLS-DA), and independent sample t-testing were used to analyze sample differences. Based on a variable importance in projection (VIP) > 1 and *p* < 0.05, 23 key volatile compounds such as octanal, geranylacetone, butyl acetate, and dihydro-*β*-ionone were screened. *β*-Ionone and phenethyl acetate made the largest contribution to the aroma of *D. delavayi* after analyzing the relative odor activity value (rOAV) of the key volatile compounds and their aroma descriptors. Young *D. delavayi* fruit exhibited a prominent woody scent, while mature *D. delavayi* fruit had more intense floral and rosy aromas. The findings may lay a foundation for comprehensively developing and utilizing *D. delavayi* fruit.

## 1. Introduction

*Docynia delavayi* is a plant of the *Docynia* genus in the Rosaceae family and a plant resource with medicinal food value that is highly distributed in southwest China [1]. *D. delavayi* fruit is rich in a variety of nutrients and polyphenolic compounds that improve appetite, prevent oxidation, and lower blood glucose [2]. The fruit can be directly eaten, preserved, or processed into fruit wine and jam, among others; it is a wild fruit with great development potential [3]. While local and international studies into *D. delavayi* have mainly focused on breeding and genetic diversity [4], functional analyses of secondary metabolite bioactivities [5], coloring mechanisms [6], processing, and applications [7], studies on aroma have been scarce thus far.

Fruit aroma critically indicates fruit quality and plant breeding, and vitally impacts fruit production and processing applications [8,9]. Aldehydes, esters, terpenoids, and other volatile compounds released by fruits contribute to the production of fruit aromas. The types and quantities of these compounds depend on the fruit variety, maturity, and environmental conditions [10,11,12]. Therefore, analyzing the volatile compounds and aroma characteristics of *D. delavayi* fruit at various stages of maturity is expected to provide an understanding of the volatile compounds in *D. delavayi* fruit and their aromas.

Headspace solid-phase microextraction (HS-SPME) is a common extraction method that adsorbs volatile compounds using silica salt fibers coated with sorbents, which not only reduces sample loss but also reduces analysis time and cost [13]. HS-SPME combined with gas chromatography–mass spectrometry (GC-MS) is a convenient, highly sensitive, solvent-free, and non-toxic method commonly used for the analysis of volatile compounds in fruits of the Rosaceae family [14,15]. GC-MS can produce a large amount of data. Therefore, the information in a large data set can effectively be exploited by combining heat map cluster analysis with principal component analysis (PCA) and orthogonal partial least squares discriminant analysis (OPLS-DA) [16,17]. In addition, aroma thresholds and the relative odor activity value (rOAV) play key roles in assessing the contribution of the aroma substance to the overall aroma [18].

Herein, HS-SPME-GC-MS in combination with OPLS-DA was employed for the investigation of the key volatile compounds that differentiate young and mature *D. delavayi* fruit. Moreover, key volatile flavor compounds were identified using odor-threshold and rOAV methods, and the differences in the aromas of young and mature fruit were compared to offer a research basis for comprehensively evaluating *D. delavayi* fruit quality and utilization.

## 2. Materials and Methods

### 2.1. D. delavayi Samples

*D. delavayi* fruit was collected from Lancang County, Yunnan Province, China (22°29′55″ N, 100°14′59″ E). Three disease- and insect-pest-free fruit trees of the same age and with similar growth were selected for sample collection. Ten pieces of *D. delavayi* fruit of similar size, shape, and maturity were collected from each tree (total of 30 samples). Voucher samples were deposited in the Collaborative Innovation Center of Forest Resource Breeding and Utilization in Yunnan, Southwest Forestry University, under accession number DY2019053. Plant species were identified by Assoc. Prof. Jianghua Liu (College of Forestry, Southwest Forestry University). The collected young and mature fruit were mixed separately, 10 pieces of fruit were randomly selected and used as biological replicates, and three biological replicates were used for each maturity level. The chopped small pieces of *D. delavayi* fruit were frozen in liquid nitrogen, and were maintained at −80 °C for use in subsequent experiments.

### 2.2. HS-SPME Conditions

Samples were taken out of the −80 °C freezer and crushed using zirconia beads in a mixing mill (MM 400, Verder Shanghai Instruments and Equipment Co., Ltd., Shanghai, China) at 30 Hz for 1.5 min before any experiment. *D. delavayi* volatile compounds were extracted using 50/30 μm DVB/CAR/PDMS fibers (Supelco, Bellefonte, PA, USA). An automatic injection method was used. (PAL RTC 120, Agilent Technologies, Santa Clara, CA, USA).

The sample (100 mg), saturated sodium chloride solution (2 mL), and n-alkanes (C_7_-C_27_, Sigma-Aldrich, St. Louis, MO, USA) (10 μL) were mixed in a 20 mL headspace vial and heated at 60 °C for 10 min, followed by exposure to SPME fibers (aged at 250 °C for 30 min before use). The volatile compounds were finally volatilized at the inlet for 5 min.

### 2.3. GC-MS Conditions

An Agilent 7890B GC system coupled with an Agilent 7000D mass spectrometer (Agilent Technologies, Santa Clara, CA, USA) was used to identify the volatile compounds in the various samples using an Agilent DB-5MS capillary column (30 m × 0.25 mm × 0.25 μm) and helium (purity > 99.999%) as the carrier gas at a flow rate of 1 mL/min. The front injection temperature and inlet mode were set to 250 °C and splitless. The GC oven temperature was set to 40 °C for 1 min, and then increased to 280 °C at 5 °C/min. EI energy of 70 eV was used during MS, the ion source and quad temperatures were at 230 °C and 150 °C, respectively. Full-scan acquisition mode (from 30 to 350 aum) was used with a solvent delay time of 3.0 min.

### 2.4. Qualitative and Quantitative Analysis of GC-MS

Acquired mass spectra were compared with spectra in the National Institute of Standards and Technology Library (NIST 14. L). Retention index (RI) values were calculated for each compound and compared with the RI values on the website. Matching volatile compounds with RI > 80 were retained, and the relative contents of the identified compounds were confirmed by dividing individual peak areas by the total peak area. The calculation of RI was performed by the expression: RI = 100 × [*n* + (Rt_(x)_ − Rt_(*n*)_)/(Rt_(_*_n_*_+1)_ − Rt_(*n*)_)], where Rt_(x)_ denotes the corrected retention time (RT) of the tested compound; *n* and *n* + 1 denote the number of carbon atoms in the previous and next n-alkane, respectively; Rt_(*n*)_ and Rt_(*n*+1)_ represent the RT of n-alkanes with *n* and *n* + 1 carbon atoms, respectively; R_(*n*)_ < R_(x)_ < R_(*n*+1)_.

### 2.5. Calculating rOAV

rOAV often serves for the assessment of the effect on the overall aroma of individual volatile compounds [19]. The rOAV of the volatile flavor compound that most obviously affected the overall flavor of the sample was set to 100. Compounds with rOAV ≥ 1 are considered critical volatile aroma compounds of the sample, and compounds with 0.1 < rOAV < 1 are considered to have a modifying effect on the flavor of the sample [20]. rOAV values were calculated using the expression: rOAV = 100 × (C_i_/C_max_) × (T_max_/T_i_), where C_i_ represents the peak area of the target volatile compound, and C_max_ represents that of the compound with the highest odor activity value; T_i_ is the odor threshold in air and T_max_ is the largest odor threshold in air.

### 2.6. Statistical Analyses

Experimental data are in the form of means ± standard deviations (SDs). Excel software (Excel 2016, Addinsoft, New York, NY, USA) and OriginPro 2022 (Origin Lab Corporation, Northampton, Massachusetts, USA) were used to collect data. IBM SPSS Statistics 26 (SPSS, Chicago, IL, USA) was used to Z-score-process all peak areas and for independent sample t-testing. TBtools (version 1.0986) was used to create heat maps. SIMCA 14.1 (Umetrics, Malmö, Sweden) was used for the PCA and OPLS-DA analyses.

## 3. Results

### 3.1. Volatile Compounds in D. delavayi Fruit with Different Maturities

**Figure 1:**
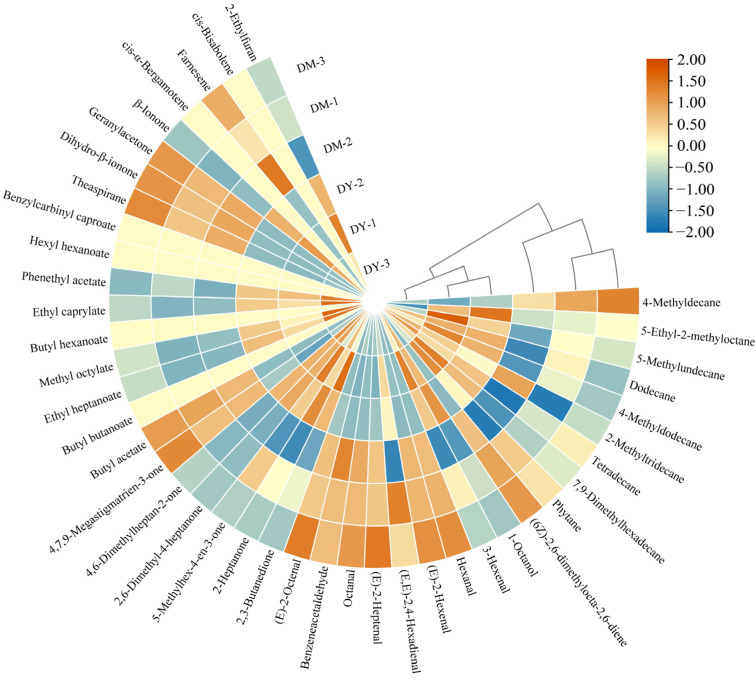
Heat maps of volatile compounds and their relative contents in *D. delavayi* fruit of varying maturities.

The volatile compounds in the samples were measured by HS-SPME-GC-MS. A total of 36 compounds were detected in young fruit (DY), including alkanes (9), an alkene (1), an alcohol (1), aldehydes (8), ketones (6), esters (5), terpenes (5), and a furan (1), while there were 42 compounds in the mature fruit (DM), including alkanes (9), an alkene (1), an alcohol (1), aldehydes (8), ketones (6), esters (9), terpenes (7), and a furan (1) (Figure 1 and Table 1).

**Figure 2:**
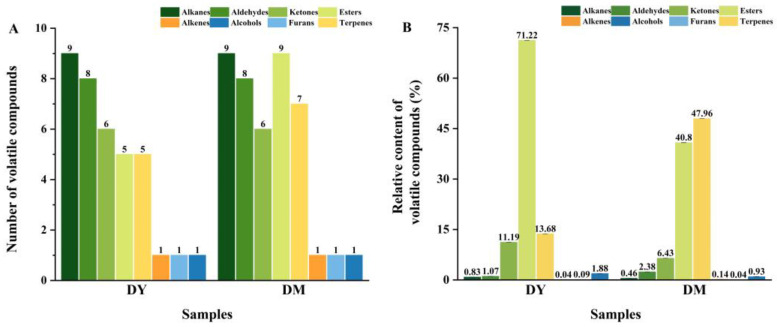
Comparing the volatile compounds in *D. delavayi* fruit of varying maturities. (**A**) Number of volatile compounds, and (**B**) relative content of volatile compounds.

The DY and DM groups displayed an obviously different number of volatile compounds (Figure 2a). Among the 42 volatile compounds, DM contained unique volatile compounds that included butyl butanoate, butyl hexanoate, hexyl hexanoate, benzylcarbinyl caproate, cis-*α*-bergamotene, and cis-bisabolene. The relative contents of the volatile compounds in DY and DM are shown in Figure 2B. Alkanes mainly originate from the homogeneous decomposition of fatty acid alkoxy radicals [14]. The alkane contents of DY and DM were 0.83% and 0.46%, respectively; however, the alkanes were observed to have a high threshold, which indicated that they hardly affected the flavor of *D. delavayi* fruit. In addition, the alkene contents in DY and DM were also relatively low (0.04% and 0.15%, respectively), as observed in other Rosaceae fruits [21]. Interestingly, small amounts of 1-octanol and 2-ethylfuran, with green aromas, were detected in both DY and DM.

Aldehydes are oxidation products of unsaturated fatty acids and a category of volatile compounds commonly found in fruits, such as tomatoes, oranges, and apples [22,23]. In this study, the number of aldehydes in both DY and DM was 8, while the contents of aldehydes were 1.07% and 2.38%, respectively, among which both DY and DM exhibited the highest (*E*)-2-hexenal contents (0.39% and 1.49%, respectively). The average ketone contents accounted for 11.19% and 6.43% of the volatile compounds identified in DY and DM, respectively. Among them, the levels of 2,6-dimethyl-4-heptanone, 2,3-butanedione and 2-heptanone were highest in both DY and DM, with each exhibiting a green aroma and a sweet fragrance.

Esters are critical fruit-aroma compounds and are mainly formed by esterifying acids with alcohols [24]. As shown in Figure 2, the number of esters in DY was fewer than that in DM (5 and 9, respectively); however, the total content of esters in DY was higher than that in DM (71.22% and 40.80%, respectively), with relatively high phenethyl acetate and ethyl caprylate contents. Phenethyl acetate is aromatic; its low threshold and high content significantly affect the overall flavor of DY and DM. Terpenes, which are considered to form through the oxidative cleavage of carotenoids, are among the most important groups of volatile compounds and play irreplaceable roles in determining the characteristic aromas of berries as well as essential roles in the phytopharmacological properties (e.g., anti-inflammatory and anticancer) of the fruit [25,26]. In the present study, terpenes accounted for 13.68% and 47.96% (on average) of the identified volatiles in DY and DM, including theaspirane, dihydro-*β*-ionone, geranylacetone, *β*-ionone, cis-*α*-bergamotene, farnesene, and cis-bisabolene; both sets of fruit contained theaspirane as the dominant terpene. Apart from *β*-ionone, DY exhibited lower terpene content than DM. Moreover, theaspirane, with a mixture of fruity and woody aromas, was the most abundant terpenoid in both DY and DM (12.19% and 34.9%, respectively).

Taken together, the volatile components of the two fruit samples differed in type and content; hence, the data were further analyzed to identify the key aromas that differentiate them.

### 3.2. Key Volatile Compounds in D. delavayi Fruit of Varying Maturities

**Figure 3:**
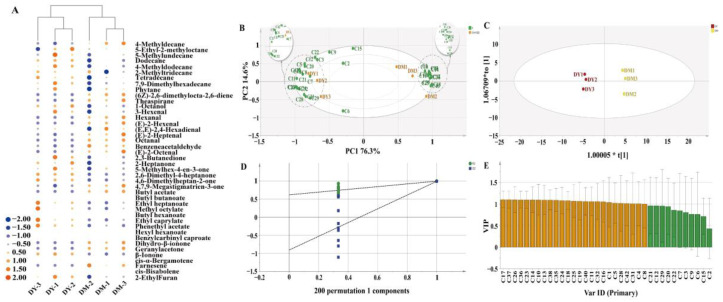
(**A**) Cluster heat map analysis. The size and color of each circle provide information on the corresponding volatile compounds in *D. delavayi* fruit samples. Blue corresponds to downregulation, orange corresponds to upregulation, and the circle size represents the degree. A blank means a lack of enrichment. (**B**) PCA biplot. (**C**) OPLS-DA score plot: *R*^2^*X* = 0.908, *R*^2^*Y* = 0.997, *Q*^2^ = 0.99. (**D**) cross-validation plot for the OPLS-DA model with 200 calculations in a permutation test: *R*^2^ = (0.0, 0.636), *Q*^2^ = (0.0, −0.878). (**E**) VIP scores. Orange corresponds to compounds with VIP > 1 and green represents compounds with VIP < 1.

A cluster heat map was produced and colored according to the relative expression change (Z-score) following the normalization of the volatile compounds in *D. delavayi* fruit samples (Figure 3a). Overall, the volatile compounds in DY were mainly upregulated, while those in DM were mainly downregulated. Twenty two volatile compounds in DY (mainly ketones and esters) were revised upwards, while 14 volatile compounds (mainly aldehydes) were revised downwards. Meanwhile, 16 volatile compounds in DM (mainly aldehydes) were revised upwards, while 20 volatile compounds (mainly alkanes) were revised downwards. These 36 volatile compounds form the basis for the different aroma characteristics of DY and DM. The key volatile compounds in DY and DM were also shown to significantly differ when column clustering analysis was used. The normalized peak areas of the 36 volatile components in the cluster heat map were subjected to PCA (Figure 3b). Principal components 1 (PC1) and 2 (PC2) accounted for 76.3% and 14.6% of the total variation (90.9%), respectively. In addition, the volatile compounds were mainly distributed in two regions. Most alkanes, ketones, and esters were located on the left side of the vertical scale, in similar positions to DY, whereas most terpenoids and aldehydes were located on the right side of the vertical scale, in similar positions to DM. Moreover, no overlap was observed between DY and DM, which indicated that their volatile components were clearly distinguishable.

**Figure 4:**
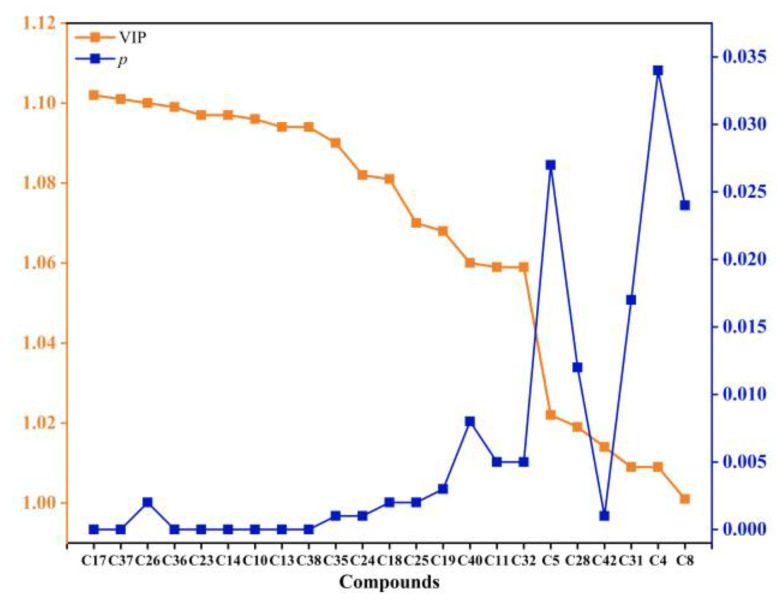
Line plots of VIP and *p* of key volatile compounds based on VIP > 1 and *p* < 0.05.

OPLS-DA serves as a new multivariate statistical method to conduct regression modeling on multiple independent variables and is capable of accurately determining the key variables impacting a group [27], and was adopted for further screening of the key volatile compounds affecting the aroma characteristics of young and mature *D. delavayi* fruit. As shown in Figure 3c, DY and DM have entirely separated score plots, with excellent fit parameters (*R*^2^*X* = 0.908, *R*^2^*Y* = 0.997, *Q*^2^ = 0.99) that confirmed the model’s accuracy. The model was further verified using 200 cross-validations. According to the test outcome [*R*^2^ = (0.0, 0.636), *Q*^2^ = (0.0, −0.878)], the OPLS-DA model did not undergo overfitting (Figure 3d). Variable importance in projection (VIP) can be used to quantify the contribution of each OPLS-DA variable to the classification; variables with VIP > 1 played vital classification roles [28]. In this study, the OPLS-DA model was developed for the identification of 25 volatile compounds with VIP > 1 (Figure 3e), with 23 key volatile compounds that satisfied VIP > 1 and *p* < 0.05 finally identified from among these 25 volatile compounds (Figure 4 and Table 2).

### 3.3. rOAV Analysis of Key Volatile Compounds in D. delavayi Fruit of Varying Maturities

**Figure 5:**
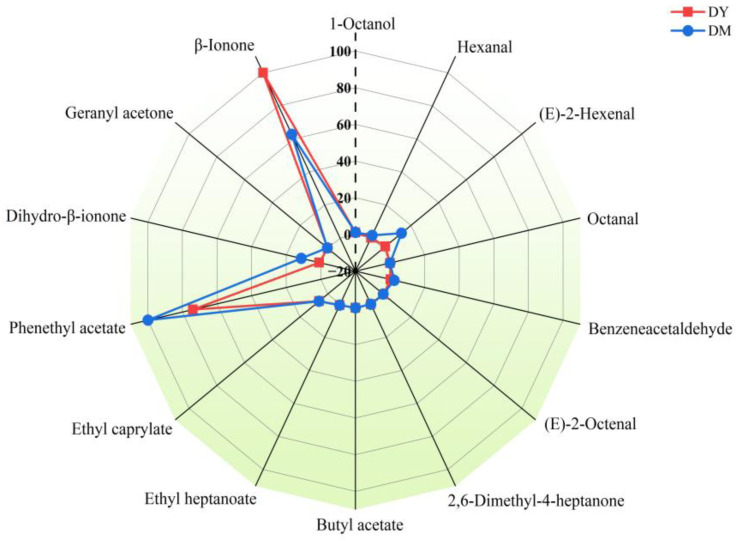
rOAV radar map of volatile aroma compounds.

rOAV data for the 14 volatile aroma compounds identified among the 23 key volatile compounds are shown in Figure 5, from which *β*-ionone and phenethyl acetate were found to mostly contribute to the overall flavor of DY and DM, respectively. Except for *β*-ionone and (*E*)-2-octenal, all compounds in DM exhibited higher rOAV values than those in DY; this discrepancy might be the reason why DY lacks a pronounced aroma.

*β*-Ionone is a 9,10/9′,10′ cleavage product of *β*-carotene, and can be extensively found in fruits, flowers, and vegetables [29]. *β*-Ionone has a low threshold; hence, the compound presents a strong woody and floral aroma at low levels. *β*-Ionone is receiving increasing levels of attention in the biomedicine field owing to its anti-inflammatory, antibacterial, and anticancer properties [30]. Phenethyl acetate is a high-value natural volatile ester with an intense floral aroma; it is the primary aromatic ester in roses, jasmine, and other plants, and has been widely added to cosmetics, food, and beverages to enhance aroma and flavor [31,32]. As shown in Table 3, volatile aroma compounds that exhibit green odors, such as (*E*)-2-hexenal (green), 1-octanol (green, citrus), and hexanal (green, grass), showed stronger rOAV in DM than in DY, which indicated that DM has a greener aroma. It is worth noting that compounds with pleasant aromas, such as benzeneacetaldehyde (sweet, honey), ethyl heptanoate (fruity, sweet), and ethyl caprylate (pineapple, fruity), were more abundant in DM than DY. Dihydro-*β*-ionone has a mellow, sweet, and fresh cedar aroma; it is an attractive compound for commercial flavor and fragrance applications, and is used in the cosmetics, perfumery, food, and beverage industries [33]. While dihydro-*β*-acetone was found to be the critical volatile aroma component in DM, it only modified the fragrance of DY. This difference is possibly one of the reasons for the different fragrances of the two samples, as was also observed for hexanal and benzeneacetaldehyde.

**Figure 6:**
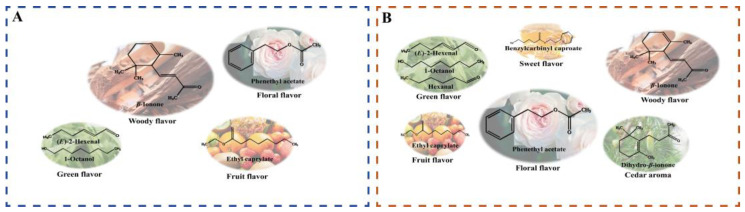
Chart showing flavor differences between young and mature fruit. The size of each ellipse only indicates the strength of each type of aroma. Main flavors in (**A**) DY and (**B**) DM.

To more intuitively analyze the differences in the aroma characteristics of DY and DM, the critical volatile aroma compounds were classified and summarized according to aroma type. Figure 6 shows that the aroma characteristics of DY and DM can be described as being woody (more prominent in DY) followed by floral and fruity. DM exhibited a more prominent floral aroma. Moreover, woody, green, cedar, fruity, and sweet aromas contributed to the more intense aroma of DM.

## 4. Discussion

Fruit aroma is an important indicator for plant breeding and influences consumer acceptance. Fruits can produce compounds that give them a characteristic aroma during the growth process; therefore, various types of volatile compounds and their quantities result in different aromas [34]. In the present study, the number of esters and terpenes in DM was more than that of DY. Esters are essential contributors to fruit aromas; they have low thresholds and generally produce pleasant odors [35]. Esters are generally synthesized through fatty acid metabolism, with critical precursors including linolenic and linoleic acids [36]; these acids are catalyzed by lipoxygenase (LOX) enzymes to generate 9-hydroperoxide (9-HPO) and 13-hydroperoxide (13-HPO) that form C6 and C9 aldehydes by the action of hydroperoxide lyase (HPL), with alcohol dehydrogenase (ADH) and alcohol acyltransferase (AAT) catalyzing their conversion to the corresponding linear esters [35].

In addition, metabolic pathways involving amino acids, such as alanine, valine, and aspartic acid, in fruit also participate in the ester synthesis [10]. Amino transferase (ATF) catalyzes the conversions of amino acids into α-ketoacids, which undergo decarboxylation, reduction, and esterification reactions to generate the corresponding aldehydes, esters, and other volatile compounds. Furthermore, α-ketoacid acids can combine with coenzyme A (CoA) to form acyl-CoA, which generates branched-chain esters by AAT catalysis [37]. Studies have shown that changes in the enzyme activities of the various synthetic pathways and sharp increases in free amino acid content during fruit ripening are possible reasons for the higher number of esters and the obvious aroma associated with fruit during ripening [21,38].

Terpenoids are the most diverse secondary metabolites and tend to have low aroma thresholds and characteristic aromas. Isopentenyl diphosphate (IPP) and dimethylallyl diphosphate (DMAPP), with C5 structures, act as the main synthetic precursors of terpenoids, with the main synthetic pathways proceeding through methylerythritol phosphate (MEP) and mevalonic acid (MVA), among which monoterpenes (C10), diterpenes (C20), and tetraterpene (C40) are mainly formed through the MEP pathway, while sesquiterpenes and triterpenes are mainly associated with the MVA pathway [26,39]. In this study, three monoterpenes (*β*-ionone, dihydro-*β*-ionone, and geranyl acetone), three sesquiterpenes (cis-*α*-bergamotene, farnesene, and cis-bisabolene), and one cyclovanilene derivative (theaspirane) were detected, with DY containing more monoterpenes and DM containing more sesquiterpenes and theaspirane. *β*-Ionone is a monocyclic monoterpene and an end-loop analog of *β*-carotenoids extensively found in fruits and vegetables, and it forms the basic structures of retinoic acid, retinol, beta-carotene, and vitamin A [40]. According to previous studies, *β*-ionone not only exhibits outstanding woody incense properties but also possesses significant pharmacological properties, including anti-inflammatory, antibacterial, and anticancer activities [30,41]. Dihydro-*β*-ionone is a monocyclic monoterpene compound with a strong cedar aroma that is often detected in *Osmanthus fragrans* flowers and tea [42,43]. Geranyl acetone is an acyclic monoterpene characterized by a rose and green fragrance; it has also been detected in blueberries and grapes [25,44,45]. Cis-*α*-bergamotene and cis-bisabolene were detected only in DM; the latter is a monocyclic sesquiterpene that exhibits antitumor, antifungal, and antimicrobial activities [46]. As an acyclic sesquiterpene, farnesene, which is an intermediate in the synthesis of vitamins E and K1, is the most abundant sesquiterpene (among cis-*α*-bergamotene, farnesene, and cis-bisabolene) [47]. C6 aldehydes, which are most common in DY and DM, are a class of compounds produced through fatty acid metabolism and mainly contribute to the green aroma; they are also critical green leaf volatiles (GLVs) [48]. Esters are the essential compounds in most ripe fruits, and aldehydes gradually become less abundant during ripening. Aldehydes are reportedly present in higher levels in fruits with more acidic and yellow peels during ripening [49], which may account for the higher aldehyde content in DM.

Human perception of fruit aroma occurs through interactions between the volatile compounds released from fruit and G protein-coupled odorant receptors (OR) in the olfactory epithelial cells of the nasal cavity. However, not all volatile compounds contribute to the aroma of the fruit, and only when the compound reaches a certain threshold will fruit aroma be affected [34]. rOAV is the ratio of the concentration of a volatile compound in the fruit to the odor threshold of the volatile compound, which assesses the promoting effect of the component on the overall fruit flavor. A higher rOAV indicates that the compound contributes more to the fruit aroma profile; this method is widely used for Rosaceae fruits [50]. The rOAV of the volatile aroma compounds was calculated to elucidate which compounds play crucial roles in regulating the overall flavor of *D. delavayi* fruit. In the present study, different rOAV scores for the same compounds in the young and mature fruit were obtained. (*E*)-2-Hexenal, *β*-ionone, 1-octanol, ethyl caprylate, and phenethyl acetate were determined to be key compounds that contribute to the overall aroma of DY, while DM also contains dihydro-*β*-ionone, hexanal, and benzeneacetaldehyde in addition to the five abovementioned key volatile aroma compounds. These findings showed that certain compounds could only decorate flavor in DY, whereas they were essential and critical compounds in DM; the overall flavor of DM was affected by more volatile aroma compounds, which may be the reason for its more pronounced odor.

*D. delavayi* is mainly produced in the Xishuangbanna and Shangri-La regions of Yunnan Province, and its fruit is commonly consumed by local ethnic minorities. *D. delavayi* has not been well promoted, and its fruit has not been well researched owing to the geography of the region. In addition, product research and development continue to focus on simple processed products, such as beverages, preserved fruits, and jams. Therefore, this study explored the differences between DY and DM in terms of the volatile components and aromas, which may effectively contribute to the comprehensive utilization of *D. delavayi* fruit.

## 5. Conclusions

The volatile components in young and mature *D. delavayi* fruit were comparatively analyzed by HS-SPME-GC-MS. DY and DM exhibited different volatile-compound profiles and aroma characteristics. Twenty three key volatile compounds, including octanal, geranylacetone, and butyl acetate, were identified using two indicators: VIP > 1 and *p* < 0.05. The rOAV of the volatile aromatic compounds revealed that DY had fewer key volatile aromatic compounds than DM, with the latter mainly exhibiting a floral aroma. Moreover, differences in young and mature *D. delavayi* fruit were analyzed from the perspective of volatile compounds and aroma characteristics. This study provides reference data for the comprehensive development, utilization, and evaluation of *D. delavayi* fruit. Furthermore, volatile compounds need to be sensory-assessed and analyzed throughout the ripening process to comprehensively understand the aroma changes exhibited by *D. delavayi*.

## Figures and Tables

**Table 1:** Volatile compounds and their relative contents in *D. delavayi* fruit of varying maturities.

NO	Classification	RT (min)	Compound	CAS	Formula	RI ^A^	Relative Content (%) ^B^	
							DY	DM
C1	Alkanes	11.69	4-Methyldecane	2847-72-5	C_11_H_24_	1021	0.03 ± 0.00	0.02 ± 0.00
C2		12.70	5-Ethyl-2-methyloctane	62016-18-6	C_11_H_24_	1054	0.09 ± 0.00	0.06 ± 0.00
C3		12.88	5-Methylundecane	1632-70-8	C_12_H_26_	1060	0.07 ± 0.00	0.03 ± 0.00
C4		17.06	Dodecane	112-40-3	C_12_H_26_	1200	0.07 ± 0.00	0.04 ± 0.00
C5		17.65	4-Methyldodecane	6117-97-1	C_13_H_28_	1221	0.04 ± 0.00	0.02 ± 0.00
C6		21.55	2-Methyltridecane	1560-96-9	C_14_H_30_	1363	0.02 ± 0.00	0.01 ± 0.00
C7		22.51	Tetradecane	629-59-4	C_14_H_30_	1399	0.12 ± 0.00	0.07 ± 0.00
C8		25.84	7,9-Dimethylhexadecane	21164-95-4	C_18_H_38_	1533	0.37 ± 0.00	0.20 ± 0.00
C9		31.20	Phytane	638-36-8	C_20_H_42_	1770	0.03 ± 0.00	0.02 ± 0.00
C10	Alkenes	12.56	(6Z)-2,6-Dimethylocta-2,6-diene	2492-22-0	C_10_H_18_	1049	0.04 ± 0.00	0.15 ± 0.00
C11	Alcohols	13.18	1-Octanol	111-87-5	C_8_H_18_O	1070	1.88 ± 0.00	0.93 ± 0.00
C12	Aldehydes	5.37	3-Hexenal	4440-65-7	C_6_H_10_O	796	0.23 ± 0.00	0.12 ± 0.00
C13		5.42	Hexanal	66-25-1	C_6_H_12_O	799	0.11 ± 0.00	0.31 ± 0.00
C14		6.72	(*E*)-2-Hexenal	6728-26-3	C_6_H_10_O	849	0.39 ± 0.00	1.49 ± 0.00
C15		8.36	(*E*,*E*)-2,4-Hexadienal	142-83-6	C_6_H_8_O	910	0.16 ± 0.00	0.11 ± 0.00
C16		9.66	(*E*)-2-Heptenal	18829-55-5	C_7_H_12_O	954	0.07 ± 0.00	0.11 ± 0.00
C17		11.08	Octanal	124-13-0	C_8_H_16_O	1001	0.01 ± 0.00	0.04 ± 0.00
C18		12.31	Benzeneacetaldehyde	122-78-1	C_8_H_8_O	1041	0.03 ± 0.00	0.15 ± 0.00
C19		12.75	(*E*)-2-Octenal	2548-87-0	C_8_H_14_O	1056	0.06 ± 0.00	0.07 ± 0.00
C20	Ketones	3.24	2,3-Butanedione	431-03-8	C_4_H_6_O_2_	680	3.76 ± 0.00	2.34 ± 0.00
C21		7.69	2-Heptanone	110-43-0	C_7_H_14_O	886	2.08 ± 0.00	1.28 ± 0.00
C22		9.73	5-Methylhex-4-en-3-one	13905-10-7	C_7_H_12_O	956	0.21 ± 0.00	0.11 ± 0.00
C23		10.09	2,6-Dimethyl-4-heptanone	108-83-8	C_9_H_18_O	968	4.97 ± 0.00	2.61 ± 0.00
C24		10.61	4,6-Dimethylheptan-2-one	19549-80-5	C_9_H_18_O	985	0.15 ± 0.00	0.08 ± 0.00
C25		27.91	4,7,9-Megastigmatrien-3-one	38818-55-2	C_13_H_18_O	1621	0.02 ± 0.00	0.02 ± 0.00
C26	Esters	5.73	Butyl acetate	123-86-4	C_6_H_12_O_2_	811	0.001 ± 0.00	0.03 ± 0.00
C27		10.85	Butyl butanoate	109-21-7	C_8_H_16_O_2_	994	—	0.11 ± 0.00
C28		13.97	Ethyl heptanoate	106-30-9	C_9_H_18_O_2_	1096	10.12 ± 0.00	4.87 ± 0.00
C29		14.72	Methyl octylate	111-11-5	C_9_H_18_O_2_	1121	0.12 ± 0.00	0.06 ± 0.00
C30		16.72	Butyl hexanoate	626-82-4	C_10_H_20_O_2_	1189	—	0.90 ± 0.00
C31		16.91	Ethyl caprylate	106-32-1	C_10_H_20_O_2_	1195	20.29 ± 0.01	9.13 ± 0.00
C32		18.57	Phenethyl acetate	103-45-7	C_10_H_12_O_2_	1254	40.69 ± 0.01	24.51 ± 0.00
C33		22.08	Hexyl hexanoate	6378-65-0	C_12_H_24_O_2_	1383	—	0.72 ± 0.00
C34		28.31	Benzylcarbinyl caproate	6290-37-5	C_14_H_20_O_2_	1639	—	0.46 ± 0.00
C35	Terpenes	20.33	Theaspirane	36431-72-8	C_13_H_22_O	1317	12.19 ± 0.01	34.90 ± 0.02
C36		23.34	Dihydro-*β*-ionone	17283-81-7	C_13_H_22_O	1432	0.13 ± 0.00	0.70 ± 0.00
C37		23.67	Geranylacetone	3796-70-1	C_13_H_22_O	1445	0.34 ± 0.00	0.60 ± 0.00
C38		24.49	*β*-Ionone	14901-07-6	C_13_H_20_O	1478	0.99 ± 0.00	0.27 ± 0.00
C39		24.76	cis-*α*-Bergamotene	18252-46-5	C_15_H_24_	1489	—	0.69 ± 0.00
C40		25.16	Farnesene	502-61-4	C_15_H_24_	1505	0.02 ± 0.00	11.38 ± 0.00
C41		27.27	cis-Bisabolene	53585-13-0	C_15_H_24_	1593	—	0.26 ± 0.00
C42	Furans	3.59	2-Ethylfuran	3208-16-0	C_6_H_8_O	699	0.09 ± 0.00	0.04 ± 0.00

^A^ Retention index. RI values were obtained from http://www.flavornet.org/flavornet.html and http://www.odour.org.uk using the same DB-5MS column (accessed on 2 November 2022). ^B^ The volatile compound content is in the form of mean value ± standard deviation (mean ± SD), “—” information was not found in the literature.

**Table 2:** Key volatile compounds based on VIP > 1 and *p* < 0.05.

NO	Compound Name	VIP	*p*
C17	Octanal	1.102	0.000
C37	Geranylacetone	1.101	0.000
C26	Butyl acetate	1.100	0.002
C36	Dihydro-*β*-ionone	1.099	0.000
C23	2,6-Dimethyl-4-heptanone	1.097	0.000
C14	(*E*)-2-Hexenal	1.097	0.000
C10	(6Z)-2,6-Dimethylocta-2,6-diene	1.096	0.000
C13	Hexanal	1.094	0.000
C38	*β*-Ionone	1.094	0.000
C35	Theaspirane	1.090	0.001
C24	4,6-Dimethylheptan-2-one	1.082	0.001
C18	Benzeneacetaldehyde	1.081	0.002
C25	4,7,9-Megastigmatrien-3-one	1.070	0.002
C19	(*E*)-2-Octenal	1.068	0.003
C40	Farnesene	1.060	0.008
C11	1-Octanol	1.059	0.005
C32	Phenethyl acetate	1.059	0.005
C5	4-Methyldodecane	1.022	0.027
C28	Ethyl heptanoate	1.019	0.012
C42	2-Ethylfuran	1.014	0.001
C31	Ethyl caprylate	1.009	0.017
C4	Dodecane	1.009	0.034
C8	7,9-Dimethylhexadecane	1.001	0.024

**Table 3:** rOAV for volatile aroma compounds in *D. delavayi* fruit of varying maturities.

NO	Compound Name	T_i_ (mg/kg) ^A^	rOAV		Odor Description ^B^
			DY	DM	
C11	1-Octanol	0.022	1.04 ± 0.07	1.15 ± 0.01	green, citrus, floral with a sweet
C13	Hexanal	0.005	0.27 ± 0.01	1.69 ± 0.20	green, grass, fruity
C14	(*E*)-2-Hexenal	0.0031	1.52 ± 0.09	13.18 ± 1.51	green
C17	Octanal	0.17	<0.1	<0.1	orange, green, herbal
C18	Benzeneacetaldehyde	0.0017	0.20 ± 0.15	2.40 ± 0.46	sweet, honey, floral
C19	(*E*)-2-Octenal	0.0027	0.28 ± 0.02	<0.1	green, herbal, banana
C23	2,6-Dimethyl-4-heptanone	9.3	<0.1	<0.1	green, fruity, pineapple, banana
C26	Butyl acetate	0.01	<0.1	<0.1	fruity, banana
C28	Ethyl heptanoate	0.24	0.52 ± 0.06	0.56 ± 0.04	fruity, sweet, banana
C31	Ethyl caprylate	0.04	6.22 ± 1.01	6.27 ± 0.52	pineapple, fruity
C32	Phenethyl acetate	0.0067	73.99 ± 4.30	100	floral rosy, sweet, honey
C36	Dihydro-*β*-ionone	0.0017	0.96 ± 0.06	11.35 ± 1.51	cedar aroma, woody mahogany aroma
C37	Geranyl acetone	0.06	<0.1	0.28 ± 0.02	rose floral, green, fruity
C38	*β*-Ionone	0.00012	100	62.78 ± 6.23	woody, floral, sweet, fruity

^A^: Ti, odor threshold: the data from compilations of odor threshold values in air, water, and other media (second enlarged and revised edition). Beijing: Science Press. 2018. ^B^: Odor descriptions adapted from http://www.thegoodscentscompany.com (accessed on 2 November 2022).

## Data Availability

The data supporting the results of this study are included in the present article.
